# The Effects of Aging on Researchers' Publication and Citation Patterns

**DOI:** 10.1371/journal.pone.0004048

**Published:** 2008-12-29

**Authors:** Yves Gingras, Vincent Larivière, Benoît Macaluso, Jean-Pierre Robitaille

**Affiliations:** Observatoire des sciences et des technologies (OST), Centre interuniversitaire de recherche sur la science et la technologie (CIRST), Université du Québec à Montréal, Succursale Centre-ville, Montréal, Québec, Canada; Université de Toulouse, France

## Abstract

The average age at which U.S. researchers receive their first grant from NIH has increased from 34.3 in 1970, to 41.7 in 2004. These data raise the crucial question of the effects of aging on the scientific productivity and impact of researchers. Drawing on a sizeable sample of 6,388 university professors in Quebec who have published at least one paper between 2000 and 2007, our results identify two turning points in the professors' careers. A first turning point is visible at age 40 years, where researchers start to rely on older literature and where their productivity increases at a slower pace—after having increased sharply since the beginning of their career. A second turning point can be seen around age 50, when researchers are the most productive whereas their average scientific impact is at its lowest. Our results also show that older professors publish fewer first-authored papers and move closer to the end of the list of co-authors. Although average scientific impact per paper decreases linearly until about age 50, the average number of papers in highly cited journals and among highly cited papers rises continuously until retirement. Our results show clearly that productivity and impact are not a simple and declining function of age and that we must take into account the collaborative aspects of scientific research. Science is a collective endeavor and, as our data shows, researchers of all ages play a significant role in its dynamic.

## Introduction

A recent study by the National Institutes of Health (NIH) [1] revealed that the average age at which U.S. researchers get their first grant from that agency has increased significantly since the beginning of the 1970s. While researchers with PhDs received their first principal investigator (PI) grant at the average age of 34.3 in 1970, this figure rose to 41.7 in 2004. This increase is also observed for PIs with MDs (from 36.7 to 43.3) as well as for those having both an MD and a PhD (from 39.3 to 43.2). Moreover, depending on the models used, it is expected that the age of new PIs could rise to 48.2 or even 54.3 in 2016. The same NIH data [2] also show that the average age of newly appointed professors in medical schools increased from 34–36 to 37.5–40 between 1980 and 2004—depending on the diploma (MD, PhD or both). This trend is not unique to the U.S. A recent study by the Association of Universities and Colleges of Canada (AUCC) [3] showed that the average age of Canadian university professors increased from 42 to 49 between 1976 and 1998 and has been stable since. These data raise the crucial question of the effects of aging on the scientific impact and productivity of researchers. Those who worry about the ageing of scientists usually believe that the younger they are the more productive they are. Better empirical data on the evolution of productivity and impact over time could thus provide important input for science policy decisions.

Since the seminal work of Lehman [4], who showed that the significant scientific contributions of researchers were generally made when they were under forty years of age, the relationship between aging and research productivity and impact has been studied extensively by both sociologists and psychologists of science [5]. However, the question of the relation between age and scientific impact or productivity remains open. Existing studies fall into two categories reporting opposite findings. The first category is based on extraordinary achievements (e.g., Nobel Prizes), finding that these tended to occur before the age of 40 [6]–[12]. Other studies in this category, measuring the productivity of scientists instead of their “creativity”, also find that younger researchers are more productive than older ones [13]–[15]. By contrast, a different body of literature on researchers' productivity reports finding that it is the researchers in the middle of their careers and older, rather than the younger ones, who are the most productive and have greater scientific impact [16]–[21].

Corresponding to this differing empirical evidence there have been differing theoretical explanations of the link between age and productivity or impact. On the one hand, studies that show that younger researchers are more productive and have higher impact are consistent with Simonton's model of creativity [22]–[23], according to which individuals have an initial “creative potential” that decreases over time. These results also make sense in view of Kuhn's argument that young researchers have a fresh look at scientific problems and are more likely to cause scientific revolutions [24]. On the other hand, research that shows that older researchers are more productive and have more impact confirms Robert K. Merton's sociological analysis that the scientific community is a *gerontocracy*, with age an important component in the stratification system of science. As they grow older, and thus gain more experience, scientists rise in the hierarchy of the scientific community, gain access to more and better resources; this, in turn, increases their productivity, impact and rewards [25]–[26].

In this paper, which is based on a much larger sample than the ones explored in previous studies, we revisit these diverging claims through measures of research productivity, scientific impact and referencing practices as a function of the of age of the researchers. In doing so, we consider productivity and impact as a collective effect of the position of researchers in the social system of science rather than the simple effect of the “creativity” of an individual [27].

## Methods

Drawing from the population of Quebec university professors and university-affiliated researchers (n = 14,469), we constructed a database containing 6,388 professors and researchers who had published at least one paper over the 8-year period (2000–2007). The average age of our publishing population (48.7 in 2003) is similar to that of Quebec professors as a whole (49.0 in 2003) [28]. In order to compile meaningful statistics data are limited to professors aged 28–70, with n≥100 university professors having published at least one paper in each age bracket. It must be noted that this study is cross-sectional. It does not follow the career of given individuals over time; it measures differences in productivity, impact and referencing patterns for professors in each age-bracket during the period 2000–2007. Hence, our sample may contain a cohort effect. Younger researchers were hired in a more competitive environment than older professors hired in the 1960s and 1970s and may be more socialized toward high productivity. There may also exist a selection bias in favor of young and highly productive researchers who would not have been hired if they had had a lower level of productivity. Despite these caveats, we think the main results and trends are robust enough to be insensitive to these possible limitations.

All indicators in this paper are constructed using bibliometric data from Thomson Reuters' Science Citation Index Expanded™ (SCIE), Social Sciences Citation Index™ (SSCI) and Arts and Humanities Citation Index™ [29], which cover about 9,000 journals annually in all fields of the natural sciences, medicine, social sciences and humanities [30]. These databases list several types of scientific documents but, as usual in bibliometric studies, we limit our analysis to articles, research notes, and review articles, which are the main forms of original publication [31]. Using, on the one hand, the surname and initials of professors and, on the other hand, the surname and initials of authors of Canadian scientific articles indexed by Thomson Reuters, a database of 115,342 articles authored by these professors and their namesakes was created. When papers were written in collaboration, we attributed one paper to each of the co-authors. In order to remove the papers authored by namesakes, each article was manually validated [32]. This time-consuming but essential step reduced the number of distinct papers by 46% to 61,857.

**Figure 1:**
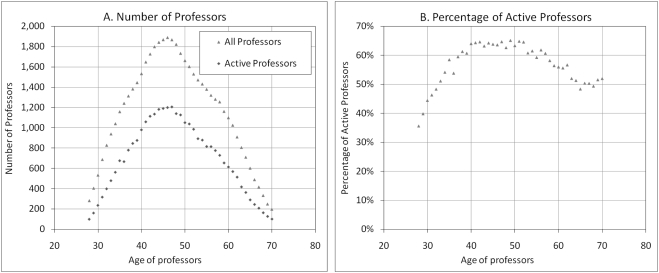
Distribution of professors by age. A) Number of professors (all professors and active professors) B) Percentage of professors who published at least one paper (active professors).

Two different sets of data are used in compiling average productivity measures. The average productivity of “all professors” counts, in its denominator, all professors that are in our sample, irrespective of whether they have published at a given age, while the average productivity of “active professors” only includes in its denominator those who published at least one paper at the age in question. Figure 1 presents the distribution of the number of professors by age (A), as well as the percentage of active professors (B). It shows that the majority of researchers (52.5% of all professors and 56.3% of the active ones) are between 40 and 55. Those younger than 40 account for 22% of all professors and 20.3% of the active ones, while those over 55 account for 25.5% of all professors and 23.4% of the active ones. As one would expect, the percentage of active professors increases steadily between 28 and 40, remains at its highest level between 40 and 55, and decreases slowly thereafter (Figure 1B).

## Results

**Figure 2:**
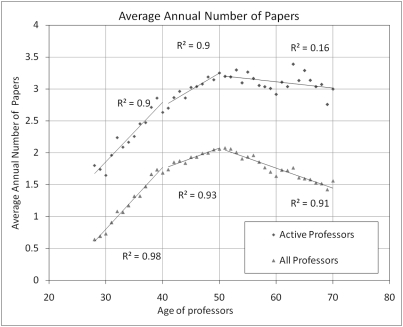
Distribution of research productivity by age. A) Average annual number of papers for professors who publish (Active) and for all professors.

Figure 2 shows the growth of the average annual number of papers per professor, with at least one paper over the period 2000–2007, using “active” professors and “all” professors as denominators. Both curves show that, between 28 and 40, research productivity increases sharply, and keeps rising at a lower pace between 41 and 50. At about age 50, productivity stabilizes for the rest of their career (for active professors) or decreases slowly (for all professors). Comparing the curves for all professors with those for the active subset shows that the latter does not drop significantly after 50, and that active professors sustain their productivity at a high level throughout their careers. Of course, only a truly longitudinal analysis following the career of a cohort of scientists during many decades could show conclusively whether those older scientists who remain highly productive are the same as those who were productive at a younger age, as some studies have suggested using smaller samples limited to a particular field [15], [17]. Our data nonetheless show clearly that active professors' productivity reaches its maximum during their fifties and tends to remain at that level until retirement. The decline observed for the all-professors curve is due to the fact that after 50, a growing fraction of professors are less active in research or have retired and stopped publishing.

**Figure 3:**
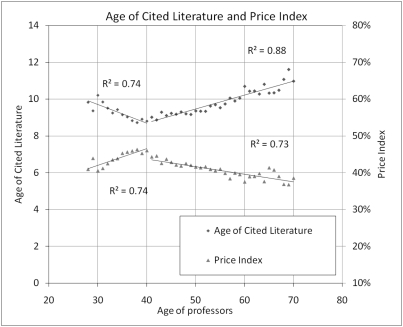
Distribution of age of cited references and percentage of those references that are 5 years or younger (Price Index for references of 100 years old or less).

Figure 3 shows the relation between researchers' age and the average age of the literature they cite. The younger the literature cited by a researcher, the likelier that that researcher is at the *forefront* of research [33]. It is striking that, again, something tends to happen around age 40. From 28 to 40, researchers cite an increasingly younger body of literature. Starting age 41, however, the literature cited ages with the author and gets older and older as time passes. This had been suggested by Zuckerman and Merton [7] and confirmed by Barnett and Fink [34]. Another way to look at this phenomenon is to compute the average *Price Index*—the percentage of cited references that are 5 years or younger [33]—for each age. Unsurprisingly, the same pattern is observed: researchers between 28 and 40 have an increasingly high *Price Index*, which steadily falls afterward until retirement. This indicator strongly suggests that the older the professors, the more distant they are from the *most recent* (forefront) scientific research. These trends suggest a simple model of scientific behavior: as young researchers rise in their productivity, they first accumulate a basic set of references in their field and add to it the most recent papers as they appear, until they are about 40. After that, a scientist tends to stick to a basic set of references and stops following the growing number of recent publications as actively.

**Figure 4:**
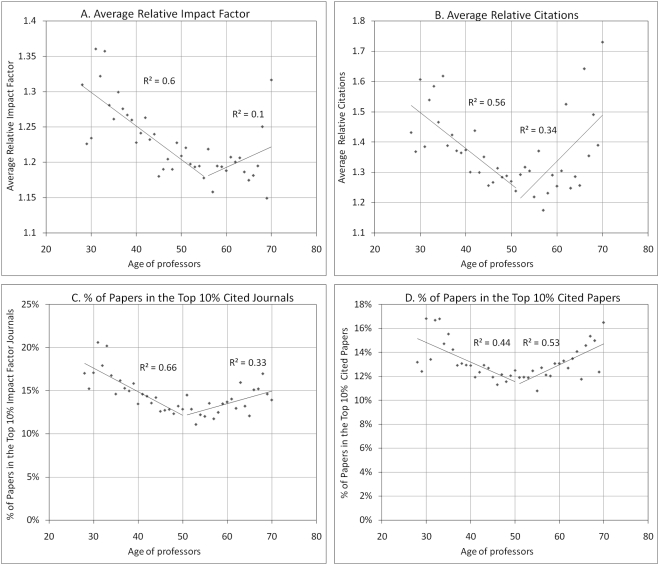
Distribution of paper research impact by professor age. A) Average relative impact factor (ARIF) B) Average relative citations (ARC) C) Percentage of papers in the top 10% cited journals D) Percentage of papers in the top 10% cited papers.

But do these turning points at age 40 and 50 also affect the scientific impact of research? To answer this question, we calculated four different indicators in Figure 4: 1) the average relative impact factor (ARIF) of the journal in which papers are published, 2) the average relative citations received by the papers over a 3-year period following publication year (excluding self-citations) (ARC), 3) the proportion of papers in the top 10% of journals ranked in decreasing impact factors (field normalized) and 4) the proportion of papers in the top 10% most cited papers (field normalized). In the calculation of the impact factors, the asymmetry between the numerator and the denominator has been corrected [36]. Also, in order to take into account the fact that publication and citation practices vary according to fields, these scientific impact measures were normalized by the world average for each subfield [37]–[38]. ARIF and ARC measures above (or below) unity mean that they are above (or below) the world average in their respective subfield. As shown in Figure 4, all impact measures show a sharp decline between 28 and about 50. However, surprisingly, impact measures subsequently rise until 70.

**Figure 5:**
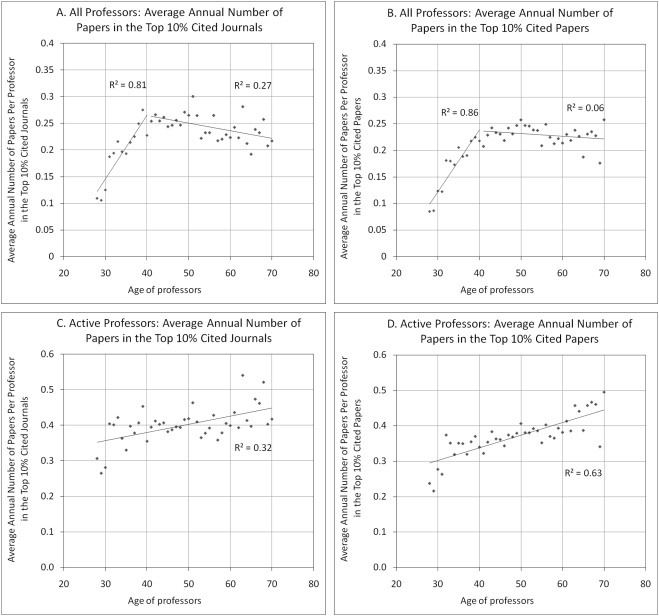
Distribution of highest impact papers by professor age. A) Average annual number of papers per professor in the top 10% cited journals (*all professors*) B) Average annual number of papers per professor in the top 10% cited papers (*all professors*) C) Average annual number of papers per professor in the top 10% cited journals (*active professors*) D) Average annual number of papers per professor in the top 10% cited papers (*active professors*).

Part of the explanation for the fact that average impact decreases between 28 and 50 and increases afterwards while productivity is rather stable for active professors can be found in Figure 5. It shows the average number of papers per researcher in the top 10% of journals ordered by decreasing impact factor and the top 10% most cited papers, using first *all professors* (A,B) and then only *active professors* (C,D). Figures 5A and 5B clearly show that there is a significant increase in the average number of papers in the top journals/papers for researchers in the range 28–40, which is rather normal given that there is also an increase in the annual number of papers for this age group. These numbers stabilize afterwards as professors publish fewer high impact papers after age 40; again, part of this decline can be explained by retirements and moves toward administrative positions. However, when only the set of active researchers is considered as the denominator (C,D), the average number of papers in the top 10% highest impact continues to rise steadily until at least 70, the oldest age for which we have at least 100 active professors in our database. Taken together, these data suggest that as they get older, researchers do not publish a lower number of *high impact* papers, but rather *dilute* these high impact papers within a larger number of lower impact papers, resulting in a decrease of their *average* impact. And given that they publish less after 50 (Figure 1) and concentrate on high impact journals and papers (Figure 5), their average impact starts to rise again (Figure 4). In summary, researchers who continue being active in research steadily increase their number of high impact papers throughout their careers.

**Figure 6:**
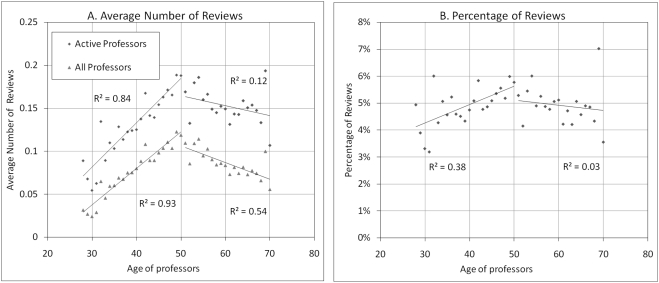
Distribution of review articles by professor age A) Average number of reviews (active professors) B) Percentage of reviews.

It might be argued that older professors have more impact because they are invited to publish more reviews, as it is known that reviews tend to get more citations than research papers [39]. Figure 6 presents, as a function of author age, the average number (A) and percentage (B) of reviews among the published papers of authors of a given age. It shows that the production of reviews by active professors increases steadily from 28 years old until 50, and gradually decreases afterwards, suggesting that there may be an optimal age to write such reviews reflecting the state of the art in a field. Similarly, the percentage of reviews among published papers increases from about 4% to 6% between age 28 and 50, and stabilizes at 5% thereafter. In light of these data, the writing of review articles cannot explain the higher productivity and impact of older professors.

**Figure 7:**
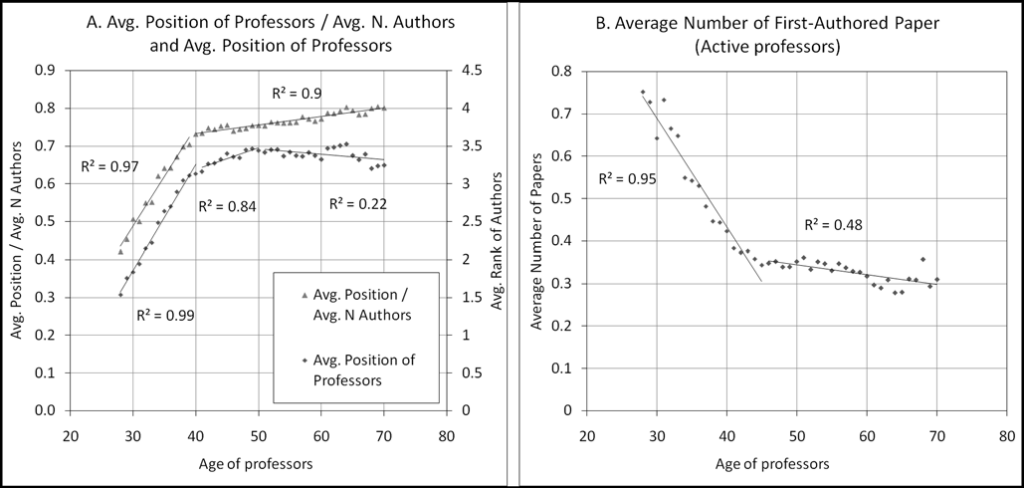
Average position of co-authors in papers by professor age. A) Average professor position and Average position divided by average number of authors per article. Papers with more than 10 authors were removed from this sample. B) Number of first-authored papers (active professors).

Another important variable that could explain our results is co-authorship, which might tend to grow with age. To measure the effect of co-authorship on older professor' productivity, Figure 7A shows, as a function of age, the average position of the authors and the ratio between their average position and the average number of co-authors This ratio can be considered a measure of the relative position of professors in the co-authors' list. It shows, as one could expect, that the average position of older professors is much closer to the end of the list. Given that the average number of co-authors per paper does not change dramatically as professors age—from 3.7 at 28 to 4.5 at 50 and then back to 4.0 at 70—the ratio between average co-authorship position and average number of co-authors increases steadily throughout the professors' career. In other words, as professors get older, they move farther away from the first position and closer to the last position in the list of co-authors. Along the same lines, Figure 7B shows that the average number of first-authored papers decreases steadily with age. Though authorship practices and the determination of co-author order vary greatly among fields [40]–[42], the first author is generally considered the main contributor to a paper. This figure shows that, as professors' rise in the hierarchy, their role in the team changes toward that of a leader and they tend to sign more as last author and less often as first author. All these results suggest that scientific productivity is not a simple function of the size of the research team, but rather of the position of professors in the hierarchy of the team, a position itself attained through accumulated experience and previous productivity.

## Discussion

The results presented in this paper show the existence of two important turning points in the research career of professors. The first happens at 40 years of age, when researchers start to rely on older literature and when the rate of increase of their productivity slows down. The second turning point is observed around 50 years old, when researchers are at the peak of their productivity while their average scientific impact is at its lowest. These data also show that older professors who stay active in research keep their productivity at a high level until their retirement. We also found that the average scientific impact of professors decreases steadily from the beginning of their careers until about 50 years old, and then increases again. Also, older professors tend to publish fewer first-authored papers and move closer to the end of the list of co-authors.

All these results can be understood in light of the changing role of researchers as they move up the stratified hierarchy of science. The fact that older researchers are more productive than younger ones clearly supports Merton's theory of cumulative advantage [25]–[26] and the “Matthew” effect [43]. Researchers who are active in their younger years gain more scientific capital [27], thereby accessing more resources, which in turn, help them stay productive, and so on. Older professors are more likely to be the leaders of their own teams, to have more resources and, hence, to sign more papers. This aspect of scientific collaboration is also consistent with the fact that they sign fewer first-authored papers than younger researchers and move their names toward the end of the list. On the other hand, the fact that younger professors have higher average scientific impact is consistent with Simonton's creativity model [22]–[23]. The fact that younger researchers are more often first authors than older ones suggests that, as team leaders, older researchers' growing impact after 50 is probably related to their building a strong team, including younger researchers [44].

In addition to these theoretical considerations, our results have science policy implications. At a time when NIH is reflecting on the role of older scientists as principal investigators [45] and some countries are re-evaluating their policy on mandatory retirement, the fact that older researchers still play an effective role in the production of high impact papers cannot be neglected. Moreover, if the turning points at 40 and 50 are relatively stable in a truly longitudinal sense, or in similar cohorts in other countries, then providing better funding opportunities to younger researchers would give them more lead time to build strong productivity before settling into a plateau. In short, as productivity and impact are not a simple and declining function of age, effective policies must take into consideration the collective aspects of scientific research and not focus on a simplistic view of “creativity” as an individualistic property of a person. Science is a collective endeavor and, as our data show, researchers of all ages play an effective role in its dynamic.
